# Ethnic and socioeconomic inequalities in relation to air pollution exposure in the Netherlands

**DOI:** 10.1093/eurpub/ckac129.563

**Published:** 2022-10-25

**Authors:** L van den Brekel, V Lenters, JD Mackenbach, G Hoek, AJ Wagtendonk, J Lakerveld, DE Grobbee, I Vaartjes

**Affiliations:** 1 Julius Center for Health Sciences and Primary Care, Utrecht University Medical Center, Utrecht, Netherlands; 2 Institute for Risk Assessment Sciences, Utrecht University, Utrecht, Netherlands; 3 Department of Epidemiology and Data Science, Amsterdam UMC, Vrije Universiteit Amsterdam, Amsterdam, Netherlands; 4 Upstream Team, Amsterdam UMC, Vrije Universiteit Amsterdam, Amsterdam, Netherlands

## Abstract

**Background:**

Air pollution (AP) contributes to a large disease burden and some populations are disproportionately exposed. It is unclear to what extent AP exposure differs across ethnic groups in the Netherlands and how this intersects with socioeconomic position (SEP). First, we identified differences in AP exposures between ethnic groups in the Netherlands. Second, we examined the interrelationships between ethnicity and SEP in relation to AP exposures.

**Methods:**

We assessed AP exposures for residents of the Netherlands in 2019 (N = 17,251,511). Home address AP levels were estimated by dispersion models of the National Institute of Public Health and the Environment (RIVM). We linked exposure estimations of particulate matter <10 or < 2.5 μm (PM10, PM2.5), nitrogen dioxide (NO2), and elemental carbon (EC) to demographic data gathered by Statistics Netherlands. Absolute and relative differences in AP levels across ethnic groups were assessed. We conducted multivariable linear regression analyses and estimated marginal mean exposures to evaluate differences by ethnicity, SEP, age and sex within urban and rural areas. We tested for interactions and stratified accordingly.

**Results:**

For the 40 largest minority ethnic groups (N > 18,314 per group), exposure to all pollutants was higher than for ethnic Dutch, with up to 1.5-fold differences for NO2. After stratification for urbanity and SEP, ethnic exposure inequalities persisted. For ethnic Dutch and some migrant groups, we found the lowest AP exposures in the middle SEP group (i.e. U-shaped trends), while we found linear patterns in other large migrant groups, with higher exposures at lower SEP.

**Conclusions:**

Exposure to PM10, PM2.5, NO2, and EC was consistently higher in minority ethnic groups compared to ethnic Dutch. The association between SEP and AP levels showed different patterns between the majority ethnic Dutch and some of the largest minority ethnic groups. Further research is needed to define the equity and health implications.

**Key messages:**

• Minority ethnic groups in the Netherlands are consistently exposed to higher levels of air pollution (PM10, PM2.5, NO2, and EC) than the ethnic Dutch population.

• Depending on the ethnic group, the association between SEP and air pollution exposure was either linear (i.e. lower exposures at higher SEP) or U-shaped (i.e. lower exposures in the middle SEP group).

